# Surgical treatment of varus unicompartmental knee osteoarthritis: indications, trends, and outcomes—a narrative review

**DOI:** 10.1007/s00402-026-06388-z

**Published:** 2026-06-18

**Authors:** Carmelo Burgio, Francesco Bosco, Claudio Cobisi, Karlos Zepeda, Fortunato Giustra, Marcello Capella, David Mayman, Eytan Debbi, Jonathan Vigdorchik

**Affiliations:** 1 Adult Reconstruction and Joint Replacement Service, Hospital for Special Surgery, New York, USA; 2 Department of Precision Medicine in Medical, Surgical and Critical Care (Me.Pre.C.C.), University of Palermo, Palermo, Italy; 3 Department of Orthopedics and Traumatology, Ospedale G.F. Ingrassia, Palermo, Italy; 4 Department of Orthopedics and Traumatology, Ospedale San Giovanni Bosco, Turin, Italy; 5 Department of Orthopedic Surgery, CTO Hospital, Turin, Italy

**Keywords:** Alignment, Unicompartmental, Osteotomy, Arthroplasty, UKA

## Abstract

**Introduction:**

Varus unicompartmental knee osteoarthritis (UC-KOA) is a frequent and heterogeneous clinical condition characterized by compartment-specific cartilage degeneration and frequently associated with lower-limb malalignment. The progressive understanding of knee biomechanics and alignment has led to the development of surgical strategies specifically tailored to address isolated compartment disease. The purpose of this narrative review is to evaluate indications, current trends, and clinical outcomes of the main surgical options for varus UC-KOA, with particular focus on realignment osteotomies and unicompartmental knee arthroplasty (UKA).

**Materials and methods:**

A narrative review of the literature was conducted using major biomedical databases. Evidence related to biomechanics, alignment correction, osteotomy techniques, UKA indications, implant evolution, complication profiles, and comparative outcomes was analyzed and synthesized thematically.

**Results:**

Biomechanical and clinical evidence consistently identifies malalignment as a key driver of isolated compartment degeneration. Corrective osteotomies effectively restore physiological load distribution and provide durable outcomes in selected patients with extra-articular deformity. Advances in implant design and surgical techniques have substantially improved UKA survivorship, allowing broader patient selection without compromising clinical outcomes. Comparative studies indicate that osteotomy and UKA serve complementary roles, with specific advantages depending on patient age, activity level, deformity origin, and joint status.

**Conclusions:**

Current evidence supports an individualized, alignment-based, compartment-specific approach to the surgical management of varus UC-KOA, emphasizing patient-specific decision-making grounded in biomechanical principles.

## Introduction

Unicompartmental knee osteoarthritis (UC-KOA) is characterized by degenerative cartilage loss and subchondral bone changes predominantly confined to a single tibiofemoral compartment—most commonly the medial compartment—while the remaining compartments are relatively preserved. This condition represents a substantial proportion of knee osteoarthritis cases and is expected to increase further as the global burden of osteoarthritis continues to rise [1, 2]. Clinically, UC-KOA typically presents with localized joint-line pain, mechanical symptoms, and mild-to-moderate varus or valgus deformity, leading to functional impairment that may still be amenable to compartment-preserving surgical interventions. The condition frequently arises from lower limb malalignment or previous joint injuries, which progressively overload the affected compartment and accelerate cartilage degeneration. Indeed, knee malalignment has been consistently identified as a central biomechanical risk factor for osteoarthritis progression, significantly influencing gait patterns and overall knee function [3, 4]. Contemporary registry data and systematic reviews have demonstrated favorable mid- to long-term survivorship for selected surgical treatments in UC-KOA. Nevertheless, failure and revision remain relevant concerns, primarily driven by disease progression in the non-treated compartments, aseptic loosening, and technical factors [5]. As a result, optimal surgical management of UC-KOA continues to represent a technical and conceptual challenge in modern orthopaedics.

Over the past decades, several surgical strategies have been developed to address this condition, most notably realignment osteotomies—such as high tibial or distal femoral osteotomy—and unicompartmental knee arthroplasty (UKA) [4, 5]. Both approaches have been shown to improve knee function, reduce pain, slow disease progression, and potentially delay or eliminate the need for total knee arthroplasty (TKA) [6, 7]. Epidemiological evidence strongly supports a compartmental approach to treatment. Recent large-scale analyses indicate that at least three-quarters of patients with knee osteoarthritis do not exhibit tricompartmental disease, a finding that contrasts markedly with the high proportion of patients currently treated with TKA. In a cohort of over 3,000 knees, single-compartment osteoarthritis was observed in approximately 50% of cases, bicompartmental disease in 33%, and tricompartmental involvement in only 17%. Isolated medial tibiofemoral, isolated patellofemoral, and combined medial tibiofemoral–patellofemoral osteoarthritis were all more prevalent than tricompartmental disease, accounting for 27%, 18%, and 23% of cases, respectively, whereas lateral compartment involvement—either isolated or combined—was comparatively less common (15%) [8]. Similar patterns have been confirmed in smaller cohorts, even among patients with advanced radiographic osteoarthritis (Kellgren–Lawrence grade > 3), where medial compartment involvement was reported in up to 91% of knees, with isolated medial disease representing the most frequent presentation [9]. Collectively, these findings support the rationale for favoring compartment-specific and joint-preserving surgical strategies over TKA whenever appropriate, with the aim of optimizing clinical outcomes in carefully selected patients.

The aim of this study is to summarize and critically discuss the current indications, evolving trends, and clinical outcomes of the main surgical options available for the treatment of varus UC-KOA, with particular focus on realignment osteotomies and UKA. By integrating Paley’s principles of deformity analysis, modern osteotomy planning strategies, and expanded indications for UKA within a unified alignment-based framework, this review proposes an original decision-making algorithm to guide patient-specific surgical planning.

## Search strategy

An extensive review of the literature addressing the surgical treatment of varus UC-KOA was performed using major biomedical databases, including PubMed, Scopus, and Web of Science. The search was limited to studies published in English and intentionally encompassed a broad range of evidence levels, consistent with the narrative and state-of-the-art nature of the review. No predefined time restrictions were applied, allowing inclusion of both seminal contributions and recent literature, up to December 2025.

The search strategy combined keywords and Boolean operators related to unicompartmental knee osteoarthritis and surgical treatment options. Key terms included “unicompartmental knee arthroplasty” OR “UKA,” “high tibial osteotomy” OR “HTO,” “distal femoral osteotomy,” “double-level osteotomy,” “varus knee osteoarthritis,” “alignment,” and “osteotomy planning”. These terms were combined in various Boolean combinations to capture literature addressing the biomechanical basis, indications, surgical techniques, outcomes, and decision-making considerations related to the treatment of varus UC-KOA.

Eligible publications included original clinical studies, randomized controlled trials, registry-based studies, systematic reviews, meta-analyses, and relevant narrative reviews addressing the surgical management of varus unicompartmental knee osteoarthritis. Case reports involving fewer than five patients, editorials without original data, and non-English publications were excluded.

The search strategy was broad and flexible, with the objective of capturing the diversity of surgical approaches, indications, technical evolution, and reported clinical outcomes related to varus UC-KOA. Because this study was designed as a narrative review, no formal risk-of-bias assessment or evidence grading was performed. The retrieved literature was screened for relevance and organized into thematic domains including: (1) lower limb alignment and biomechanics, (2) realignment osteotomies, (3) unicompartmental knee arthroplasty, (4) patient selection and surgical decision-making, and (5) complications and comparative outcomes. This narrative framework was adopted to provide a comprehensive, coherent, and up-to-date overview of current concepts, emerging trends, and areas of ongoing debate in the surgical management of varus UC-KOA.

## Biomechanics

**Fig. 1:**
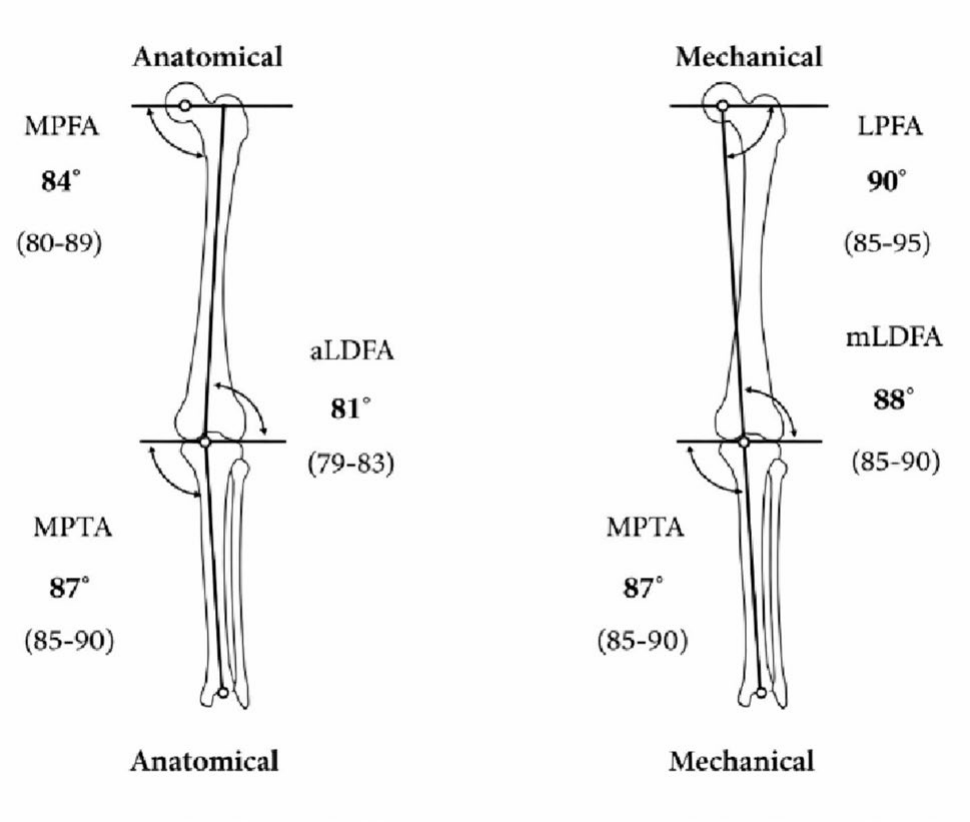
Anatomical and mechanical alignment of the lower limb. Schematic illustration of reference axes and normal angular values of the distal femur and proximal tibia, including the mechanical proximal femoral angle (MPFA), anatomical and mechanical lateral distal femoral angles (aLDFA and mLDFA), and the medial proximal tibial angle (MPTA). The source is published under a Creative Commons License from Watanabe Y, Takenaka N, Kinugasa K, Matsushita T, Teramoto T (2019) Intra- and Extra-Articular Deformity of Lower Limb: Tibial Condylar Valgus Osteotomy (TCVO) and Distal Tibial Oblique Osteotomy (DTOO) for Reconstruction of Joint Congruency. Adv Orthop 2019:8605674

Rational surgical planning in varus UC-KOA depends on accurate analysis of mechanical axes and joint-orientation angles, as originally described by Paley. A key step in evaluating lower limb malalignment is determining whether the deformity is extra-articular or intra-articular. Coronal alignment of the distal femur and proximal tibia is defined by the mechanical lateral distal femoral angle (mLDFA) and the mechanical medial proximal tibial angle (mMPTA), both of which normally range between 85° and 90° [10, 11]. When either value falls outside this range, the deformity is extra-articular – located in the distal femur, the proximal tibia, or both, - and correction should be directed at the affected bone segment through an appropriately planned osteotomy [11] (Fig. 1).

In contrast, intra-articular deformities are characterized by normal mLDFA and mMPTA values but abnormal joint congruence. These alterations are typically reflected by an increased joint line convergence angle (JLCA > 3°–4°), pathological joint-line obliquity (JLO), or femoro-tibial coronal subluxation, and usually result from asymmetric cartilage loss or ligamentous imbalance rather than true bony malalignment [12]. In such cases, realignment osteotomy does not address the underlying problem; it introduces a compensatory metaphyseal deformity that may reduce load and pain but with unpredictable effects on joint congruency and potential complications for later prosthetic replacement [13, 14]. Failure to make this distinction can lead to overcorrection, secondary deformities, and altered ligament tensioning, underscoring that precise differentiation between extra-articular and intra-articular origins is essential to guide appropriate surgical treatment [12–14].

## Osteotomy

Varus malalignment associated with medial unicompartmental knee osteoarthritis (UC-KOA) is commonly treated with valgus high tibial osteotomy (HTO) or distal femoral osteotomy (DFO) [15]. Osteotomy is typically indicated in young or middle-aged active patients with preserved range of motion and isolated medial compartment degeneration. In appropriately selected patients, osteotomy aims to redistribute joint loading and delay arthroplasty while preserving the native joint [15, 16]. Additional indications include combined femoral and tibial deformities, sagittal plane abnormalities (posterior tibial slope alterations), and selected cases of ligamentous instability [15–17].

### Osteotomy techniques

Osteotomies around the knee can be performed using monoplanar or biplanar configurations. Monoplanar osteotomies involve a single osteotomy plane and are primarily used to correct coronal malalignment while preserving the native posterior tibial slope [15, 18]. They are generally suitable for mild-to-moderate deformities and cases without significant sagittal plane abnormalities.

**Fig. 2:**
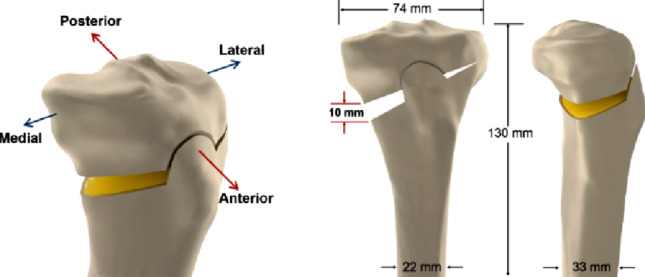
Schematic illustration of proximal tibial biplanar osteotomy geometry and anatomical orientation. The source is published under a Creative Commons License from Yang JC, Lin KY, Lin HH, Lee OK (2021) Biomechanical evaluation of high tibial osteotomy plate with internal support block using finite element analysis. PLoS One 16(2):e0247412

Biplanar osteotomies incorporate an additional ascending or descending cut, increasing the bone contact area and improving rotational stability. This configuration also allows better control of sagittal alignment and posterior tibial slope, which is particularly relevant in the presence of ligamentous insufficiency. For example, slope-reducing osteotomy may benefit ACL-deficient knees by reducing anterior tibial translation, whereas slope-increasing osteotomy may improve stability in PCL-deficient knees [19, 20]. Because of these advantages, biplanar techniques are commonly preferred when simultaneous coronal and sagittal correction is required (Fig. 2).

### Single-level vs. double-level osteotomy

When deformity arises predominantly from a single bone segment, correction should be performed at that anatomical site, consistent with modern deformity-correction principles [17]. Tibial deformities (MPTA < 87° with normal mLDFA) are typically treated with HTO, whereas femoral deformities (mLDFA > 88° with normal MPTA) require DFO.

In some patients, deformity originates from both the distal femur and proximal tibia or a single-level correction would produce excessive joint line obliquity (JLO). In such cases, double-level osteotomy (DLO), combining femoral and tibial correction, may be indicated to restore physiological alignment while maintaining a horizontal joint line [19, 22]. Reported indications include bifocal deformity, excessive predicted MPTA (> 94–95°), JLO > 4–5°, or large mechanical axis deviation (> 9–10°) in young active patients. Recent series suggest that DLO can achieve more physiological postoperative alignment without increasing complication rates in appropriately selected patients [15, 19, 21, 22].

### Planning methods

Preoperative planning is based on standing full-length radiographs to determine mechanical axis deviation and the site of deformity. Historically, correction targets were defined using the Fujisawa point, corresponding to approximately 62.5% of the tibial plateau width, producing slight valgus alignment [16, 23]. Contemporary strategies increasingly emphasize patient-specific correction targets based on cartilage status, meniscal integrity, ligament balance, and patient factors such as age and activity level. Consequently, the desired postoperative weight-bearing line is often planned between 50% and 62.5% of the tibial plateau width, with many surgeons targeting approximately 55% to avoid excessive valgus correction.

Several planning methods have been described, including the Dugdale and Miniaci techniques. The Dugdale method provides a simple and widely used approach for estimating correction angles, whereas the Miniaci method incorporates hinge geometry and may provide more precise anatomical correction [18, 24, 25]. More recently, the New Mikulicz–Joint Line (NM-JL) method has been proposed for double-level osteotomies, allowing simultaneous femoral and tibial correction while maintaining physiological joint-line orientation [22]. Regardless of the method used, accurate identification of the deformity origin and careful preoperative planning remain essential for achieving durable mechanical correction.

## Unicompartmental knee arthroplasty

UKA represents an established surgical option for the treatment of osteoarthritis confined to a single knee compartment, most commonly the medial compartment [26, 27]. Introduced in the 1970s, UKA was conceived to address symptomatic unicompartmental disease in patients not yet suitable for TKA, offering the theoretical advantages of preserving near-physiological knee kinematics while reducing surgical invasiveness, blood loss, and postoperative recovery time compared with TKA [28]. Despite these advantages, early and mid-term studies reported relatively high revision rates—approaching 28% at 10 years in some series—thereby prompting a critical reappraisal of implant design, surgical technique, and patient selection criteria [29]. Over time, this led to substantial refinement of indications and technical concepts, supporting the progressive shift toward minimally invasive and patient-specific UKA strategies.

### Indications and patient selection

The first widely accepted indication criteria were proposed by Kozinn and Scott in 1989 and reflected the conservative approach of the era: low-demand patients older than 60 years, body weight below 82 kg, osteoarthritis or osteonecrosis confined to a single compartment, minimal pain at rest, a preoperative ROM greater than 90°, flexion contracture less than 5°, and angular deformity less than 15° that was passively correctable to neutral alignment [29]. These criteria contributed to improved early results by restricting UKA to a narrow, low-risk population, but they also excluded a substantial number of patients who might have benefited from a compartment-preserving strategy.

### Contemporary expanded indications

Subsequent large-cohort studies and meta-analyses have progressively challenged several of the original restrictions. Systematic review data indicated that increased BMI does not consistently compromise implant survival or pain relief, with long-term survivorship rates ranging from 69.5% to 90.9% at 20 years even in overweight and obese patients [31–33]. Female patients appear to achieve superior survivorship compared with males at 10-, 15-, and 20-year follow-up, potentially reflecting differences in activity patterns, whereas no consistent survival difference has been demonstrated between patients younger or older than 60 years at the time of surgery [31]. Recently, single-center data lend further support to this trend. In a propensity-matched comparison of 70 octogenarians and 70 younger patients undergoing medial RA-UKA, Innocenti et al. reported comparable improvements in KSS and OKS scores at a mean follow-up of 36 months, with both groups exceeding thresholds for clinically meaningful change and no differences in implant survival or complication rates [30]. However, the study was limited by a small sample size with low statistical power for detecting between-group differences, a mid-term follow-up that precludes conclusions about long-term survivorship, and a selection process that likely favored healthier octogenarians. These findings suggest that advanced age alone need not exclude patients from consideration for UKA, though they should be interpreted with caution until confirmed by larger, longer-term, multicenter studies. Conversely, some evidence suggests a higher likelihood of revision in younger and more physically active individuals [32, 33].

### Role of ligament status and patellofemoral joint involvement

Traditional contraindications for UKA have also been critically re-evaluated. Historically, ACL deficiency and patellofemoral joint (PFJ) degeneration were considered major risk factors for implant failure. However, contemporary evidence indicates no significant differences in functional outcomes or revision rates between patients with and without PFJ osteoarthritis [34]. In contrast, ACL deficiency remains a relevant factor influencing polyethylene wear and progression of degeneration in the lateral and patellofemoral compartments. Selected clinical studies suggest that one-stage combined ACL reconstruction and UKA may result in improved implant survivorship compared with UKA performed in ACL-deficient knees without ligament reconstruction [35–37].

### Fixation methods: cemented versus cementless UKA

Regarding fixation, cemented UKA remains the most adopted technique in current clinical practice [38]. Comparative studies assessing cemented and cementless fixation have not demonstrated significant differences in revision rates or complication profiles at mid-term follow-up, although some authors report a higher incidence of complete pain relief with cemented implants [38, 39].

### Implant design: fixed-bearing versus mobile-bearing

**Fig. 3:**
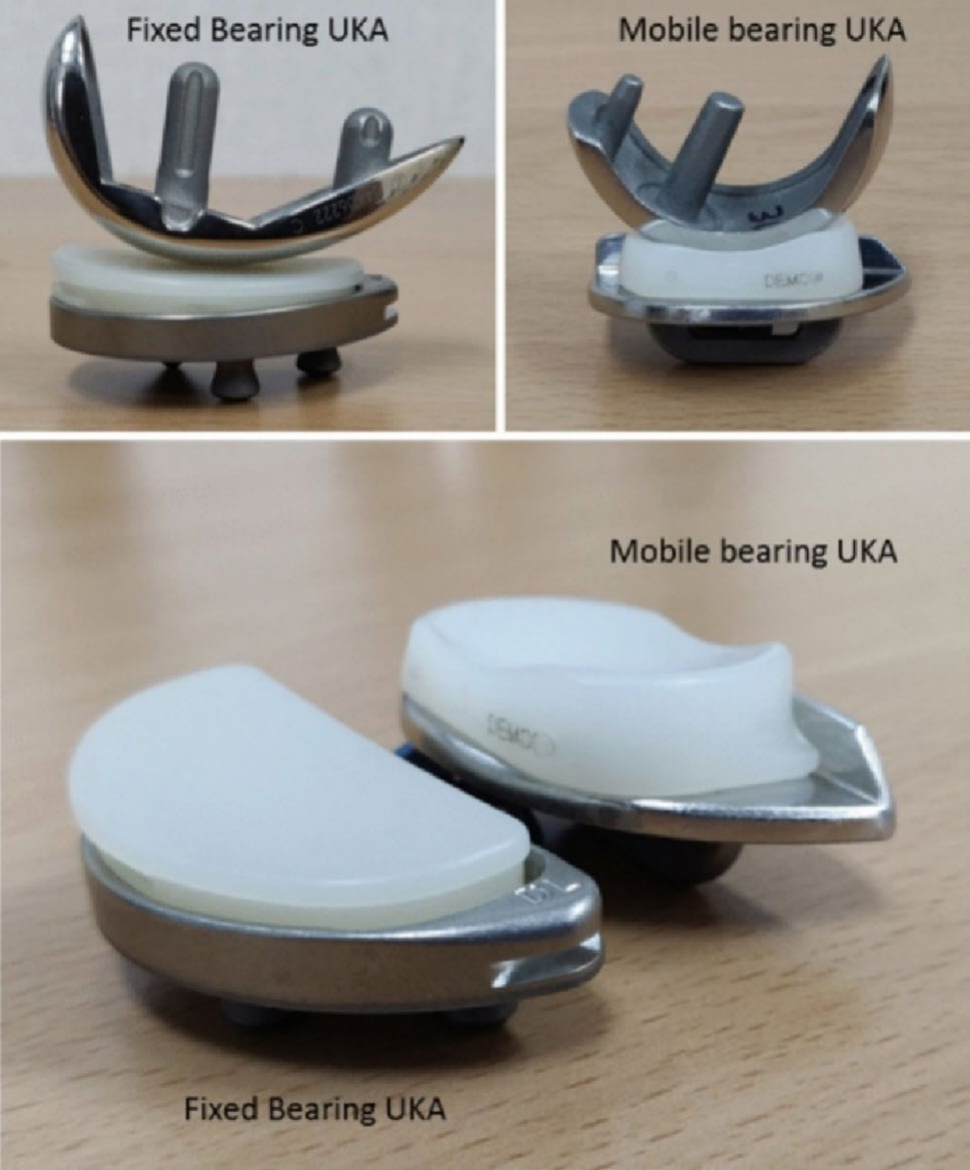
Fixed-bearing and mobile-bearing unicompartmental knee arthroplasty (UKA) designs. The source is published under a Creative Commons License from Mittal A, Meshram P, Kim WH, Kim TK (2020) Unicompartmental knee arthroplasty, an enigma, and the ten enigmas of medial UKA. J Orthop Traumatol 21(1):15

UKA implants are generally categorized as fixed-bearing or mobile-bearing designs (Fig. 3). Mobile-bearing implants were developed to enhance knee kinematics, reduce contact stress, and minimize polyethylene wear [40]. Randomized controlled trials have shown no significant differences in clinical outcomes—such as WOMAC and Oxford Knee Scores—between the two designs. Nevertheless, biomechanical and simulator studies consistently report increased polyethylene wear in fixed-bearing implants due to reduced conformity, findings that are supported by in vivo investigations [39, 41, 41]. Although some series report higher patient satisfaction with mobile-bearing UKA, overall implant survivorship appears comparable between fixed- and mobile-bearing designs [39–41].

### Robotic-assisted UKA

Technological advances have further expanded interest in UKA, particularly through the adoption of robotic-assisted techniques. Available evidence suggests that robotic UKA may improve component positioning accuracy and limb alignment. Gilmour et al. reported that more active patients may derive greater benefit from robotic-assisted (RA) UKA, although long-term survivorship data remain limited [42]. A recent meta-analysis by Ghazal et al. demonstrated that RA-UKA systems achieve improved hip–knee–ankle alignment and higher Oxford Knee Scores compared with conventional techniques, while emphasizing the need for further high-quality comparative studies [43]. Similarly, comparative analyses of different robotic platforms have reported improved radiographic accuracy, although clear superiority in long-term clinical outcomes has yet to be established [44]. Of note, the safety and functional effectiveness of RA-UKA across a wide age range, including octogenarians, has recently been reported with comparable mid-term outcomes between elderly and younger cohorts, though these data remain preliminary [30].

Two practical considerations temper the current enthusiasm for RA-UKA. First, the learning associated with robotic platforms is not negligible. The literature demonstrates variable learning curves ranging from 20 to 40 cases to achieve consistent operative times and to fully exploit the accuracy advantages of the system, with early cases often associated with longer operative duration without corresponding improvements in radiographic outcomes [45, 46]. Second, the cost-effectiveness of RA-UKA remains poorly defined. The capital cost of robotics in the existing literature has not convincingly demonstrated that improvements in component positioning translate into reductions in revisions rates sufficient to offset these costs at a population level [47, 48]. Until longer-term data clarify whether radiographic precision afforded by robotic assistance produces measurable clinical and economic benefits, the adoption of these platforms should be weighed against institutional resources and case volume [49].

## Patient selection and decision algorithm

**Fig. 4:**
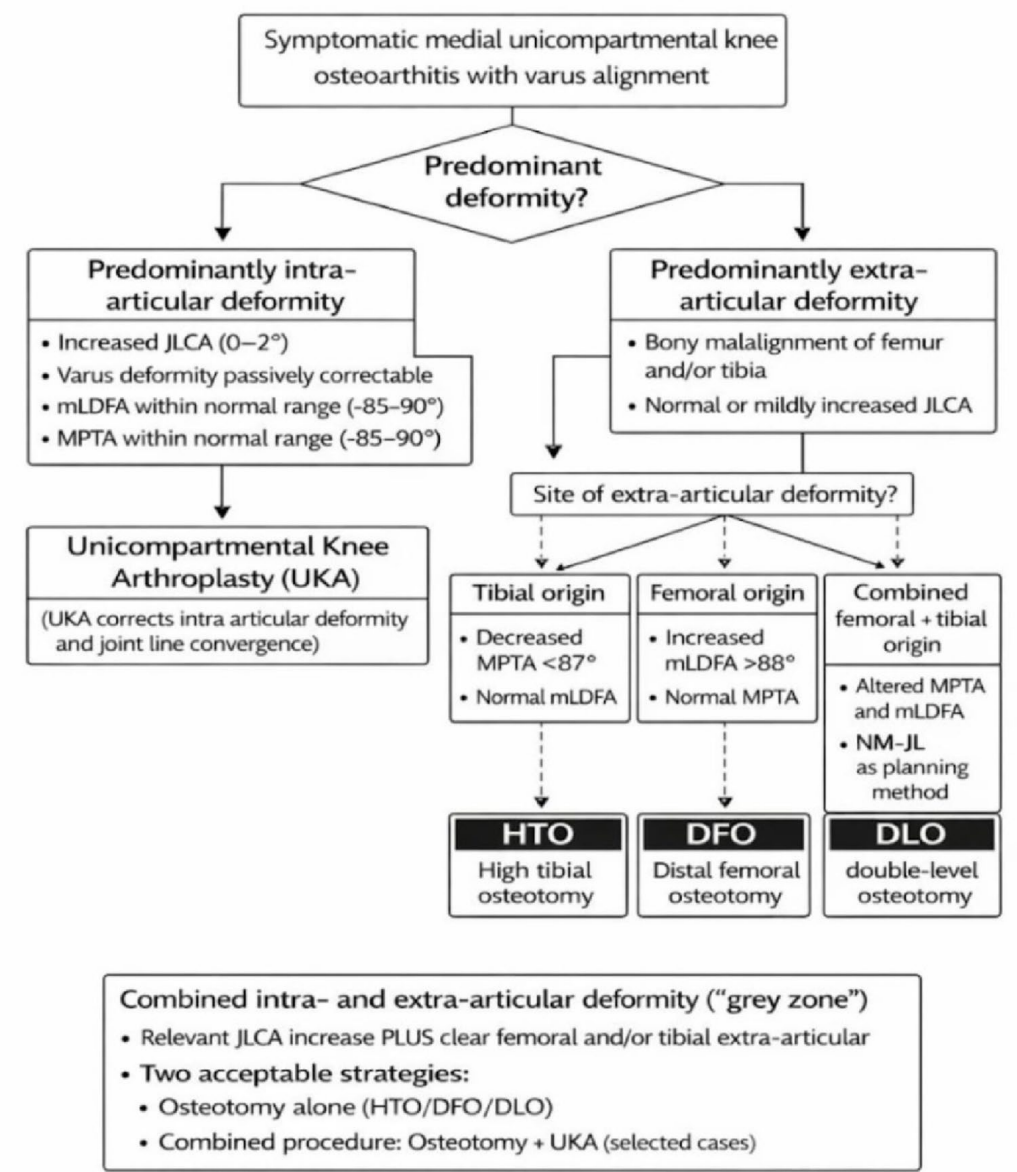
Decision-making for the surgical management of varus medial unicompartmental knee osteoarthritis. UKA, unicompartmental knee arthroplasty; HTO, high tibial osteotomy; DFO, distal femoral osteotomy; DLO, double-level osteotomy; JLCA, joint line convergence angle; mLDFA, mechanical lateral distal femoral angle; MPTA, medial proximal tibial angle; NM-JL, New Mikulicz–Joint Line.

A fully standardized and universally accepted decision-making algorithm for the surgical treatment of varus UC-KOA is not yet available. To address this gap, we propose an original alignment-based decision algorithm (Fig. 4) that synthesizes the evidence reviewed in the preceding sections into a practical clinical framework. The algorithm integrates Paley’s deformity analysis, current osteotomy indication criteria, and contemporary UKA selection principles into a stepwise pathway linking deformity origin, compartment status, and patient characteristics to a specific surgical strategy [50, 51].

Based on current evidence, UKA is generally indicated in symptomatic patients with medial UC-KOA who are aged ≥ 60 years with Kellgren–Lawrence (K–L) grade ≥ 3, or in patients younger than 60 years with K–L grade 4 disease. Additional prerequisites include functional cruciate and collateral ligaments, a correctable varus deformity not exceeding 10°, and the absence of clinically relevant lateral or patellofemoral compartment pathology. Importantly, UKA is best conceptualized as a procedure that primarily addresses the intra-articular component of varus alignment (e.g., asymmetric cartilage loss and ligamentous imbalance), while extra-articular deformity should be corrected at its bony origin with an osteotomy [50, 51]. Conversely, HTO is preferentially indicated in symptomatic patients aged ≤ 70 years with medial compartment osteoarthritis graded K–L 2–3, varus malalignment of tibial origin (MPTA 87° ± 3°) and a varus deformity ≥ 5°, if body mass index (BMI) is < 35 kg/m². In cases where indications for UKA and HTO overlap, shared decision-making is recommended, with detailed discussion of advantages and limitations of each procedure [52].

Within an alignment-based framework, the anatomical origin of extra-articular varus should further drive the osteotomy choice. When varus deformity is predominantly tibial, correction is typically achieved with proximal tibial osteotomy, most commonly medial opening-wedge HTO, whereas other strategies (e.g., lateral closing-wedge) may be considered depending on the planned correction and surgeon preference. When the deformity is predominantly femoral (i.e., varus of distal femoral origin), a valgus DFO should be considered to correct the extra-articular component at the appropriate level and to avoid non-physiological joint-line orientation caused by “forcing” a tibial correction in a femoral-driven deformity. Finally, when extra-articular varus deformity is bifocal (both femoral and tibial), a varus DLO may be indicated to distribute correction between the two segments, restore the global mechanical axis, and preserve a physiological joint-line orientation [22]. According to Goodell et al., HTO is particularly suited for younger patients (< 55 years), with BMI < 30 kg/m², high activity demands, mechanical malalignment with asymmetric varus, isolated ACL insufficiency, the need for multiplanar correction, and a preference for joint-preserving strategies [52]. Importantly, more recent evidence [51–53] suggests that chronological age alone (> 55 years) should not represent an absolute contraindication to HTO. In contrast, UKA may be favored in patients older than 55 years with lower activity levels, BMI < 40 kg/m², advanced osteoarthritis with significant joint-space narrowing, acceptable coronal alignment, symmetric varus deformity, and patient preference for arthroplasty [53]. Earlier studies suggest that the ideal candidate for HTO is a young (< 60 years), active patient affected by symptomatic mild-to-moderate varus knee (5–15°) with mild medial compartment involvement (e.g., less than grade III, Ahlbäck classification), intact lateral and patellofemoral compartments, good knee ROM (knee flexion > 120°), and no joint laxity or instability [52–53]. Over time, the indications for HTO have been expanded to include posterolateral laxity and varus hyperextension thrust, ACL deficiency associated with varus alignment, and combined ligamentous laxity with varus or posterolateral thrust, while obesity remains a controversial factor [53–55].

Indications for UKA have similarly evolved and currently include UC-KOA or femoral condyle avascular necrosis, with intact lateral and patellofemoral compartments, age over 60 years, low demands, no obesity, minimal pain at rest, ROM over 90° of flexion with less than 5° flexion contracture, and within 10° of axial malalignment, which can be passively corrected almost to neutral [55, 56]. In selected “gray-zone” scenarios characterized by combined intra-articular degeneration and clinically relevant extra-articular deformity, a combined strategy (e.g., osteotomy to correct the extra-articular component and UKA to address intra-articular wear) has been described. However, this approach remains uncommon and supported mainly by limited clinical evidence and highly selected cases; therefore, it should be reserved for carefully selected patients and experienced centers [53–56] (Fig. 4).

## Complications and failures

### Complications and failures after knee osteotomy

Osteotomies around the knee—particularly medial opening-wedge HTO and DFO—are effective joint-preserving procedures but are associated with a relevant rate of mechanical, biological, and hardware-related complications.

A large systematic review by Miltenberg et al. including 7,836 patients reported intraoperative and postoperative complication rates of 5.5% and 6.9%, respectively. The most frequent intraoperative event was lateral hinge fracture (LHF) (9.1%), while postoperative complications included superficial infection (2.2%), non-union (1.9%), loss of correction (1.2%), and implant failure (1.0%). The overall reoperation rate reached 15.5%, mainly due to hardware removal [57]. In medial opening-wedge HTO, LHF represents the most clinically relevant complication. Franulic et al. reported LHF in approximately 6% of cases and implant failure in 8.2%, with unrecognized fractures leading to delayed union or correction loss [58]. Koh et al. demonstrated a significantly increased LHF incidence when the opening gap exceeded 10 mm and showed that unstable fracture patterns were associated with inferior early outcomes [59].

The biomechanical and prognostic significance of LHF has been extensively clarified by the classification proposed by Takeuchi et al. [58]. According to this system, Type I fractures extend through the lateral cortical hinge at or above the level of the proximal tibiofibular joint and are generally considered mechanically stable. Type II fractures propagate distally toward the proximal tibiofibular joint, compromising the lateral buttress and reducing construct stability. Type III fractures extend proximally into the lateral tibial plateau and represent the most unstable configuration, often associated with intra-articular involvement. Clinical and biomechanical studies consistently demonstrate that Type II and Type III fractures are associated with significantly increased micromotion at the osteotomy site, higher rates of delayed union or non-union, loss of correction, and fixation failure compared with Type I fractures. Consequently, accurate intraoperative recognition of hinge fracture type is critical to guide fixation strategy, postoperative weight-bearing protocols, and the need for additional stabilization [59, 60].

Complications are not limited to tibial osteotomies. In lateral closing-wedge DFO, Rupp et al. reported medial hinge fractures in 48% of cases, with malunion occurring in 14% when hinge displacement exceeded 2 mm [61]. Overall, the most critical complications of knee osteotomies include hinge-zone fractures, delayed union or non-union, loss of correction, and hardware-related reoperations, which may reach 15–25% in long-term series [57, 61].

### Complications and failures after UKA

Failure mechanisms following UKA have been extensively evaluated in contemporary systematic reviews and registry-based studies. Migliorini et al. reported revision rates of 6–8% at 5 years and 10–15% at 10 years, most commonly due to osteoarthritis progression (25–35%), aseptic loosening (20–30%), and malalignment-related failure (10–20%) [1]. Registry data corroborate these findings, with Obermayr et al. reporting a five-year revision rate of 7–8% and a significantly higher risk among low-volume surgeons [62].

Outcomes after conversion of failed UKA to TKA are generally favorable. Pumford et al. demonstrated five-year survivorship rates of 92–94%, with low rates of re-revision, stiffness, and infection [63]. Compared with revision TKA, revision UKA is associated with higher implant survivorship, reduced need for constrained components, and lower major complication rates, as shown by Forlenza et al. [64]. Overall, although UKA presents specific modes of mechanical failure, its revision pathway is typically less complex and less morbid than that of revision TKA.

An important caveat when evaluating UKA outcomes is the well-documented discrepancy between national registry data and single-center or designer series. Registry-based studies consistently report higher revision rates than series originating from high-volume centers with established expertise in UKA [62]. Several factors contribute to this gap: registries capture the full spectrum of surgical volume and experience, including low-volume surgeons for whom revision risk is demonstrably higher, whereas single-center series typically reflect optimized patient selection, standardized technique, and dedicated follow-up protocols. Reporting bias and loss to follow-up further complicate direct comparison. When citing survivorship figures, it is therefore important to distinguish between the two evidence of sources and to recognize that outcomes achievable in specialized settings may not be directly generalizable to broader practice.

## Comparative evidence HTO versus UKA

HTO and UKA are both established treatments for isolated medial compartment osteoarthritis, yet they differ in their postoperative profiles and are best suited to different patient populations. Rather than competing strategies, they should be understood as complementary procedures whose relative strengths depend on the clinical context.

### Functional outcome

Available meta-analytic data indicate that UKA tends to provide superior early pain relief and higher WOMAC scores compared with HTO [53]. Hoorntje et al., in a multicenter analysis of patients aged 50–60 years with Kellgren–Lawrence grade 3–4 disease, reported slightly higher Oxford Knee Scores and patient satisfaction after UKA, although the differences did not reach statistical significance [65]. These findings are consistent across several comparative series, suggesting a modest but reproducible advantage for UKA in early patient-reported outcomes. Conversely, HTO has been associated with greater postoperative ROM in some comparative studies [53]. This may be particularly relevant for younger, physically active patients who prioritize return to demanding activities, though direct evidence linking improved ROM after HTO to superior long-term activity levels remains limited.

### Revision and survivorship

Short-term revision rates have been reported to favor HTO in some series, while others show lower early revision risk after UKA – a discrepancy likely driven by differences in patient selection, surgical volume, and the definition of revision endpoints across studies [50, 53]. At longer follow-up, survivorship appears comparable when each procedure is performed within its optimal indication range. Kim et al. reported no difference in survival between UKA (96%) and medial open-wedge HTO (98%) when performed within their respective indication ranges [50]. Functional recovery, measured by Knee Society, Lysholm, and Tegner scores, does not consistently favor either procedure [53].

### Conflicting evidence and the gray zone

The most significant area of uncertainty involves patients aged between 50 and 65 years with K-L grade 3 medial OA and moderate varus malalignment – a population in which both HTO and UKA can be reasonably justified [51, 52]. For these patients, the available literature offers no definitive answer. Observational studies point in different directions depending on the outcome measure, follow-up duration, and selection criteria used. Whether UKA or HTO produces better long-term results in this subgroup remains genuinely unresolved, and the current evidence base is insufficient to support a firm recommendation for one over the other. Shared decision-making, guided by deformity origin, activity expectations, and patient preference, is the most defensible approach until higher-quality comparative data becomes available.

## Discussion

The present review provides a comprehensive, alignment-based overview of the contemporary surgical management of varus UC-KOA. The principal contribution of this work is the alignment-based decision algorithm presented in Fig. 4, which synthesizes deformity analysis, contemporary osteotomy planning strategies, and expanded UKA indications into a practical framework for surgical decision-making. Within this framework, varus UC-KOA is recognized as a heterogeneous condition in which osteotomy and UKA may represent appropriate alternatives to total knee arthroplasty in selected patients, depending on deformity origin, compartment involvement, and patient characteristics.

A consistent body of biomechanical and clinical evidence supports lower-limb malalignment as a major driver of isolated compartment degeneration. Varus alignment increases medial tibiofemoral contact pressures and medial meniscal extrusion, thereby accelerating cartilage breakdown and disease progression in medial UC-KOA [5, 39, 66, 67]. In this context, the distinction between extra-articular deformity and intra-articular deformity is fundamental, as it directly influences whether correction should be achieved through osteotomy or arthroplasty. Failure to identify the true origin of malalignment may lead to non-physiological joint-line obliquity, ligament imbalance, and inferior long-term outcomes [5, 65, 67, 68].

In this review, we described the two opposite ends of the treatment spectrum: corrective osteotomy in the presence of predominant extra-articular deformity with relatively preserved joint surfaces, and UKA in cases of advanced intra-articular degeneration with minimal constitutional malalignment. In practice, however, many patients fall between these two extremes, with coexisting intra-articular degeneration and extra-articular deformity. In these cases, treatment selection should not rely on rigid historical criteria alone, but rather on the relative predominance of each deformity component and the broader clinical context.

Contemporary evidence supports the continued role of HTO and DFO as effective joint-preserving procedures in appropriately selected patients, with durable improvements in pain and function and acceptable mid- to long-term survivorship [4, 66, 69, 70, 71]. More recently, increasing attention to joint-line orientation and double-level osteotomy has refined correction strategies for bifocal deformities, allowing restoration of global alignment while preserving more physiological postoperative joint-line orientation [19, 21, 22, 68].

UKA has also evolved substantially. Improved implant design, fixation methods, and patient selection have expanded indications beyond the classical Kozinn–Scott criteria, particularly with respect to age, BMI, and limited patellofemoral degeneration [29–33]. Registry and cohort data support favorable long-term outcomes in well-selected patients, although revision rates remain consistently higher in national registries than in single-center or designer series, likely reflecting differences in surgical volume, experience, and patient selection [5, 42, 62, 67]. Comparative evidence suggests that UKA generally provides superior early pain relief and faster recovery, whereas osteotomy may offer greater flexibility in younger, highly active patients and in those with multiplanar deformity or associated ligamentous insufficiency [50, 53, 65].

Technological innovations have further refined both treatment modalities. Modern locking plate systems have improved the stability and reproducibility of osteotomies, while advancements in UKA implant geometry and bearing design have enhanced kinematic restoration. Robotic-assisted UKA has demonstrated improved accuracy in component positioning and limb alignment, with early evidence suggesting potential functional benefits, particularly in active patients [72]. However, robust long-term data confirming a clear clinical superiority over conventional techniques are still lacking, and cost-effectiveness remains a relevant consideration [72, 73].

Complication profiles also differ between procedures and must be carefully weighed during decision-making. Osteotomies are associated with specific risks, including hinge fractures, nonunion, loss of correction, and hardware-related reoperations, with reported reoperation rates reaching 15–25% in long-term series [57–61]. Conversely, the most common causes of UKA failure include progression of osteoarthritis, aseptic loosening, and malalignment-related issues, with revision rates of approximately 10–15% at 10 years [4, 62]. Nevertheless, conversion of failed UKA to TKA generally yields favorable outcomes and is less complex than revision TKA, further supporting UKA as a viable compartment-specific option in selected patients [63, 64].

### Clinical implications

On the basis of the evidence reviewed, several practical points warrant emphasis. First, preoperative planning should begin with systematic evaluation of deformity origin, as the distinction between extra-articular and intra-articular pathology is the key determinant of treatment selection. Second, osteotomy should be performed at the anatomical site of deformity to avoid non-physiological correction and excessive joint-line obliquity. Third, modern UKA indications are broader than traditional historical criteria and should not be restricted solely on the basis of age or body mass index. Finally, in patients who fall within the overlap between osteotomy and UKA indications, shared decision-making remains essential and should incorporate deformity pattern, activity level, ligament status, and patient expectations.

### Controversies and unresolved questions

Several areas remain unresolved. The optimal treatment for patients aged 50–65 years with moderate varus deformity and intermediate osteoarthritis severity remains uncertain, as available comparative evidence is largely observational and sometimes conflicting. The long-term performance of double-level osteotomy relative to single-level correction requires further study, particularly outside high-volume centers. Similarly, although robotic-assisted UKA improves radiographic precision, it remains unclear whether this translates into durable clinical or economic benefit. Finally, the role of combined osteotomy and UKA in selected “gray-zone” patients with both extra-articular deformity and advanced intra-articular degeneration remains supported only by limited evidence.

## Limitations

This review has several limitations inherent to its narrative design. No formal quality assessment or risk-of-bias evaluation of the included studies was performed. As a narrative review, the strength of evidence was considered qualitatively rather than through formal grading, which may limit the ability to weigh individual findings according to their methodological rigor. In addition, the absence of predefined search strategies and quantitative synthesis introduces potential selection bias. Additionally, substantial heterogeneity across included studies—regarding patient populations, surgical techniques, implant designs, fixation methods, follow-up duration, and outcome reporting—limits direct comparability and precludes definitive conclusions.

Furthermore, much of the available evidence originates from high-volume, specialized centers, which may reduce external validity when extrapolated to general clinical practice. Several clinically relevant topics—including lateral UKA indications, long-term outcomes of double-level osteotomies, the role of robotics, and combined intra- and extra-articular correction strategies—remain supported by low-level or inconsistent evidence. The lack of standardized complication reporting further limits accurate risk stratification.

Future prospective, multicenter studies with standardized outcome measures are therefore needed to refine patient selection algorithms and to establish evidence-based guidelines for the optimal surgical management of UC-KOA.

## Conclusions

Varus unicompartmental knee osteoarthritis is a heterogeneous condition in which limb alignment and compartment involvement critically influence surgical decision-making. Current evidence supports an alignment-based, compartment-specific approach rather than routine total knee arthroplasty. Corrective osteotomies and unicompartmental knee arthroplasty represent complementary strategies, with osteotomy favored in younger, active patients with extra-articular deformity, and unicompartmental knee arthroplasty offering reliable pain relief and predictable outcomes in patients with isolated intra-articular disease. Accurate preoperative assessment of deformity origin and ligament status is essential to optimize outcomes. Advances in implant design and surgical technology have expanded indications for both procedures, reinforcing the need for individualized, patient-specific treatment algorithms.

## Data Availability

No datasets were generated or analysed during the current study.
